# Retinal Vessel Width Measurement at Branchings Using an Improved Electric Field Theory-Based Graph Approach

**DOI:** 10.1371/journal.pone.0049668

**Published:** 2012-11-27

**Authors:** Xiayu Xu, Joseph M. Reinhardt, Qiao Hu, Benjamin Bakall, Paul S. Tlucek, Geir Bertelsen, Michael D. Abràmoff

**Affiliations:** 1 Department of Biomedical Engineering, University of Iowa, Iowa City, Iowa, United States of America; 2 Department of Electrical and Computer Engineering, University of Iowa, Iowa City, Iowa, United States of America; 3 Department of Ophthalmology and Visual Science, University of Iowa, Iowa City, Iowa, United States of America; 4 Veteran's Administration Medical Center, Iowa City, Iowa, United States of America; 5 Department of Community Medicine, University of Tromsø, Tromsø, Norway; University of Tennessee, United States of America

## Abstract

The retinal vessel width relationship at vessel branch points in fundus images is an important biomarker of retinal and systemic disease. We propose a fully automatic method to measure the vessel widths at branch points in fundus images. The method is a graph-based method, in which a graph construction method based on electric field theory is applied which specifically deals with complex branching patterns. The vessel centerline image is used as the initial segmentation of the graph. Branching points are detected on the vessel centerline image using a set of detection kernels. Crossing points are distinguished from branch points and excluded. The electric field based graph method is applied to construct the graph. This method is inspired by the non-intersecting force lines in an electric field. At last, the method is further improved to give a consistent vessel width measurement for the whole vessel tree. The algorithm was validated on 100 artery branchings and 100 vein branchings selected from 50 fundus images by comparing with vessel width measurements from two human experts.

## Introduction

### Motivation

The retinal vessel width relationship at vessel branch points in fundus images is an important risk factor of retinal and systemic disease, including ischemic heart disease, hypertension and brain abnormalities, [1], [2]. The relationship between retinal arterial diameters at branch points conform to predicted optimal values in normal subjects, but deviate significantly in patients with peripheral vascular disease [3]. Studies also showed that this relationship deviates from the theoretic optimum with advancing age [4]. Increased branching coefficients of retinal vessels have been reported to be associated with periventricular white matter hyperintensities and ischaemic heart disease, and decreased branching coefficient with deep white matter hyperintensities [1]. In some conditions, such as hypertension, the smaller arteries are affected more than the larger ones [2]. Compared with using vessel width directly as a parameter, the vessel width relationship at the branch point is dimensionless, which allows measurements without correcting for the differences in magnification by the optics of the eye across individuals, caused by different refractive errors. [5], [6].

### Previous Work

Though image analysis of retinal vessel has been widely studied and over two hundreds papers have been published in the field of retinal vasculature detection and vessel width measurements [7], only a few studies focus on vessel width measurement at branch point and proposed specific methods to solve these type of problems [1], [3], [8]–[11]. In general, studies that treat branch points separately focused on two phases: a) detection of branch point, and b) vessel width measurement at branch point.

Most of the retinal branch point detection methods target using the bifurcations as a landmark for further image analysis [8]–[10], [12]–[14], such as retinal image registration. Shen *et al.* in 2001, proposed a real-time landmark extraction method from fundus images. This model-based method detects branch points as a fragment of the vasculature that consist of two relatively straight anti-parallel edges with either an intensity peak or an intensity valley in between. Tsai *et al.* further refined this method in 2004, in which the detected branch point is used as the initial estimated branch point. Then an exclusion region is provided around the estimated branch point and the location is further refined within the exclusion region. In 2008, Bhuiyan *et al.* proposed a method to detect vascular bifurcations and crossovers based on the vessel geometrical features. A binary vessel image is first segmented from the color fundus image and morphological thinning operation is applied to find the vessel centerline. Subsequently, rotational invariant 3
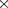
3 masks are used to detect potential bifurcations and crossover points. Finally, the geometrical and topological properties are used to refine the result. A detection accuracy of 95.82% was reported.

Compared with vessel branch point detection, fewer studies have addressed the problem of width measurement at branch points. In most retinal branching studies, the vessel width is measured by manual methods ([2], [4], [15], [16]) or semi-automatic methods ([1], [3], [11]). In a study on the relationship of peripheral vascular disease and arterial bifurcation diameter, Chapman *et al.* proposed a semi-automatic method to measure the vessel width for arteries [3]. This method needs human expert operators to draw lines perpendicular to arteries and then an automatic method is used to determine the points of maximum intensity variation based on the cross-sectional profiles [17]. This method is not specific for retinal vascular branchings. Doubal *et al.* proposed a semi-automatic method that can track down each branch if the branch center is identified by a trained grader. Then the cross-sectional profiles can be obtained and a Gaussian curve is fit to determine the width [1]. Each profile needs manual inspection after the fitting. Patton *et al.* used a similar semi-automatic method to calculate the branching vessel width [11]. The human operator manually identifies the arterial and venous branch points and draw a line perpendicular to the vessel. Four other intensity profiles can be automatically generated from the given profile. Each profile can be rejected or accepted by the operator. Then a Gaussian fitting is used to identify the vessel width.

To our knowledge, no fully automatic method has been developed to specifically deal with the vessel width measurement problem at branch points in fundus image.

## Methodology

We have previously published a graph-based method to measure the vessel width for straight vessel segments [18]. The vessel centerline image is used to obtain the base nodes for the graph. Then, a two-slice three-dimensional graph is luxated for each vessel segment. The graph columns are built perpendicular to the vessel growing directions. The perpendicular direction is calculated using principal component analysis (PCA). A smoothness constraint between the two slices is applied. Thus, the simultaneous two-dimensional boundary segmentation problem is transformed into a three-dimensional surface segmentation problem. It is further converted to a minimum closed set problem in a node-weighted graph. By solving this minimum closed set problem, the two boundaries of blood vessels can be determined and the vessel width can be measured. Because graph columns are constructed along the second principal component of PCA, this method will be referred to as the PCA-method in the remainder of this paper.

The PCA-method for straight vessel width measurement cannot be extended to the measurement of branch point directly. First, the graph columns will intersect each other as the graph columns approach the branching center, resulting in multiple vessel width measurements at the same point. Second, the graph columns might run into adjoining vessel branches, resulting in meaningless measurements of vessel width inside the vessel segment. In order to address these problems, a different graph construction method is needed. We propose an electric field theory motivated graph construction method to solve this problem. Similarly, because the graph columns are constructed along the electric lines of force, this method is referred to as the ELF-method.

### Pre-processing and Bifurcation Detection

**Figure 1:**
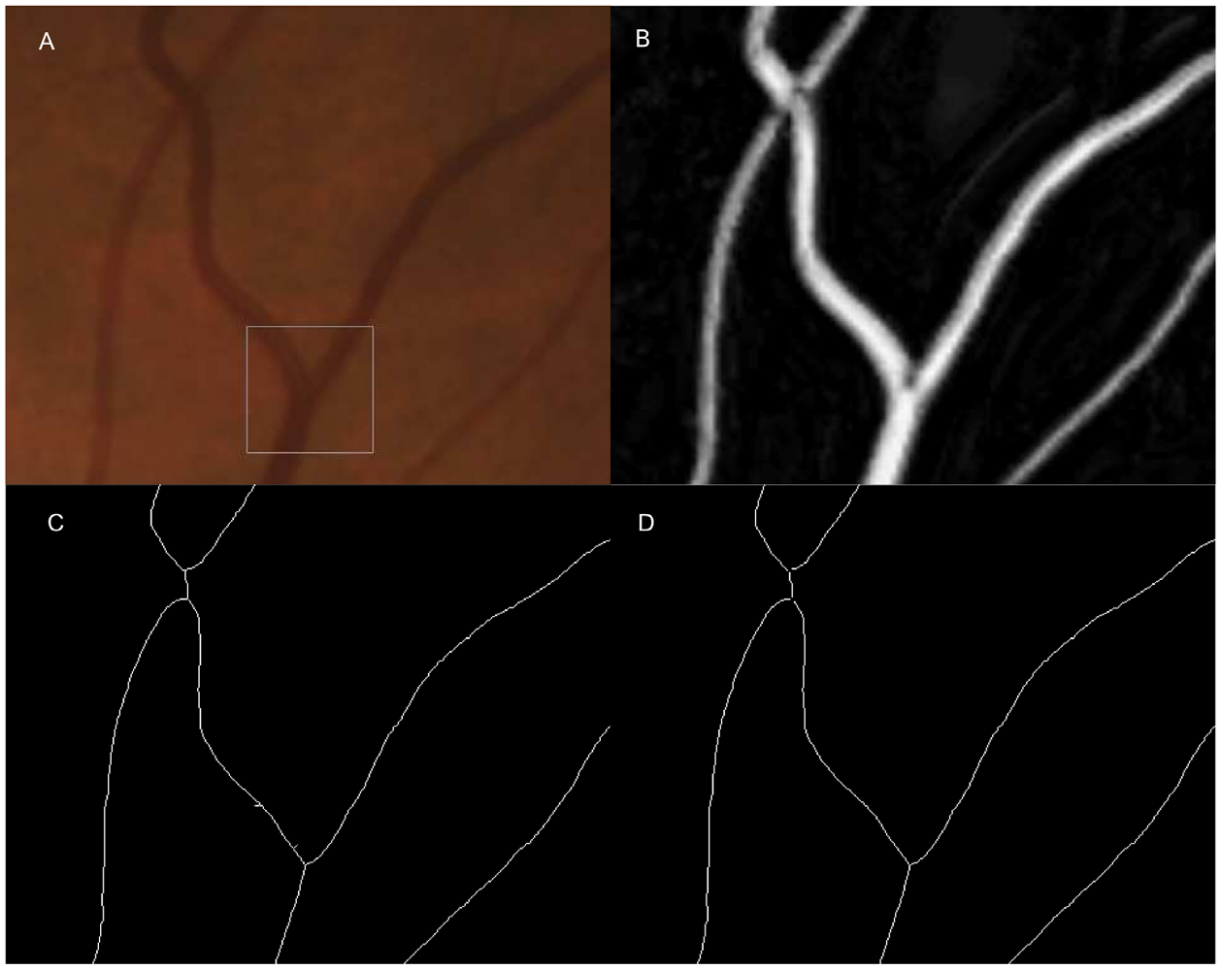
Spur pruning and crossing point exclusion on vessel centerline image. (a) An enlarged part of a color fundus image. Branch inside the rectangle indicates the branch in Figure 1. (b) The corresponding vesselness map. (c) The corresponding vessel centerline image with spur presented. A crossing point is split into two branch points. (d) The corresponding vessel centerline image after spur pruning and crossing point exclusion.

The goal of pre-processing is to obtain a vessel centerline image as the initial segmentation. We start with the vessel segmentation map as proposed by Niemeijer *et al.* in [19]. As in the original study of the vessel segmentation, we used the images and reference standard of the DRIVE database (http://www.isi.uu.nl/ Research/Databases/DRIVE/). One example image is shown in Figure 1 (b). The vessel segmentation map is a gray scale image with each pixel assigned the likelihood of being in a blood vessel, the higher the intensity, the higher the likelihood. By thresholding the vessel segmentation map, a binary vessel segmentation image is generated. A constant low threshold of 70 is chosen to better maintain the continuity of blood vessels. The trade-off is that small regions of noise may not be suppressed adequately. Thus, vessel regions with an area smaller than 20 pixels are erased from the binary vessel image. A sequential thinning approach is then applied to the binary vessel segmentation image to find the vessel centerline [20].

Branch points are detected on the vessel centerline image using a series of 3
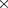
3 kernels [20]. The kernels are given in Equation 1.
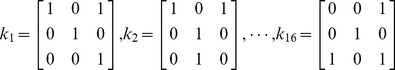
(1)


These kernels can effectively detect all branch points which are shown on the vessel centerline image. The ability for the proposed algorithm to automatically localize the correct vessel centerline was validated in Reference [18]. However, not all detected branch points are true vessel branchings, as shown in Figure 1 (c). False branches can result from abrupt vessel width changes, vessel direction changes, or from noise. Moreover, the vessel crossings are often detected as two adjacent branchings on the vessel centerline image. Hence, a spur pruning and crossing point exclusion step is applied.

Starting from a detected branch point, all three branches are traced. If another branch point is reached within certain distance, the two branch points are regarded as a single crossing point and excluded from further study. On the other hand, if an end point is reached within certain distance, the traced branch is regarded as a spur and removed from the centerline image.

### Graph-Based Vessel Boundary Segmentation

We apply an electric field theory motivated graph construction method to build the graph at branch points. This graph construction method was first proposed by Yin *et al.* in 2009 [21]. The method is inspired by the non-intersecting property of electric lines of force. Recall Coulomb's law:
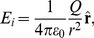
(2)where 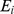 is the electric field at point 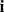, 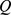 is the charge of point 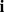, 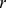 is the distance from the point 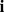 to the evaluation point 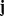, 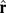 is the unit vector pointing from the point 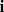 to the evaluation point 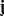, and 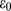 is the vacuum permittivity. The total electric field E at point 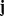 is the sum of 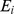:
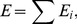
(3)the electric field has the same direction as the *electric line of force* (ELF).

When multiple source points exist in an electric field, the electric line of force holds a non-intersecting property. If we change 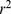 to 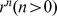, the non-intersection property still holds. The difference is that the vertices with larger distances will be penalized in ELF computing. A value of 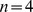 is used to decrease the effect from pixels with a larger distance and hence increase the robustness of local ELF computation.

**Figure 2:**
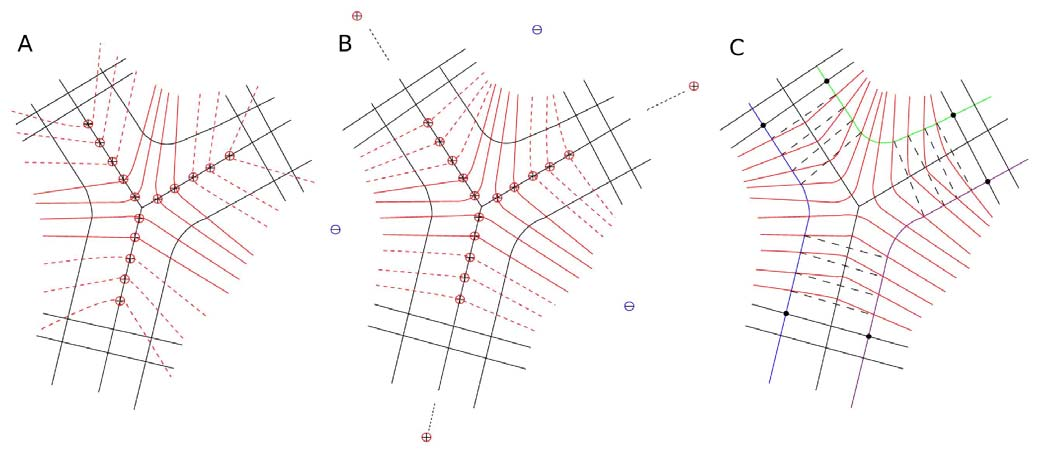
Illustration of problems in applying traditional graph construction method to branch points and introduction to electric field theory based graph construction method. (a) A figure to illustrate possible problems in applying traditional graph construction method to branch point situation. The problems include possible graph column intersection and graph columns running into another vessel branch. (b) Introduction to electric field theory based graph construction method. Its most attractive is the non-intersecting property of electric field lines.

By applying this theory to the problem of graph construction at branch points, we assume each vessel centerline pixel is a positive unit charge. The electric line of force is calculated and the graph is constructed along the electric line of force, as illustrated in Figure 2 (b).

**Figure 3:**
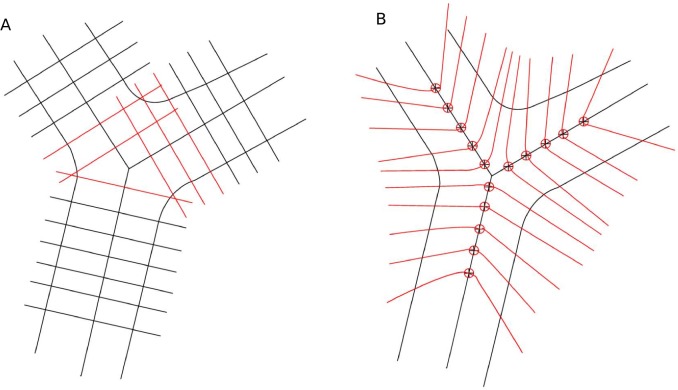
Improvement of the electric field theory based graph construction method. For illustration purposes, not all graph columns are shown in the figure. (a) Problems encountered when combining the traditional graph and electric field theory based graph. The graph columns intersect each other because the graph columns from the electric field theory based method point outside rather than the normal direction when approaching the end of branches, as shown in dashed red lines. (b) Improved combined graph. Two strategies are introduced to improve the construction of the combined graph. (c) Three graphs are built for the three boundaries separately, as shown in color green, blue, and purple. After the three boundaries are determined, the vessel width is measured as the Euclidean distance between the corresponding nodes from the same centerline pixel.

If we consider branch points to be isolated from vessel trees, the graph given above is good enough to give a reliable measurement. However, if a consistent vessel width measurement is desired for the whole vessel tree, problems will arise at the transition from the graph built using ELF-method for branch points to the graph built using PCA-method for the adjoining straight vessels, as shown in Figure 3 (a). The electric line of force points outside at the end of each branch. When the two different types of graphs are connected, the graph columns from different methods will intersect.

We propose two strategies to improve the electric field theory based graph construction method, as illustrated in Figure 3 (b). First, in order to pull the electric lines of force towards the normal direction at the end of each branch, extra positive charges and negative charges are added in the electric field. Positive charges with extremely large values are positioned at the infinite extension of each branch. This is used to generate a parallel force along the vessel growing direction that can push the graph columns toward the perpendicular direction. A distance of 10,000 pixels is used to simulate the infinite distance and the charge value was chosen to be 100,000 unit charges. Values of the distance and charge are not sensitive and do not have a large effect as long as they are big enough to be considered as “infinity” in this case. In order to further pull the graph columns towards the normal direction, three negative charges are positioned in the middle points of three branches. Values of the negative charges are also not sensitive as long as they are comparable to the value of adjacent positive charges. We chose the value to be five in this case.

Second, a combined graph construction method is introduced at the transition location. Six centerline pixels at the transition location are used to build the combined graph. First of all, the location of graph nodes are calculated using both the PCA-method and the ELF-method, given by 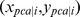 and 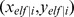, where 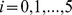 is the distance from the graph column to the branching center. Then the node locations in the combined graph are calculated using a weighted linear interpolation of 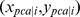 and 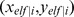:
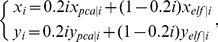
(4)where 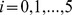.

### Cost Function

The cost function is generated from convolution of the image's green channel with an oriented first order derivative of Gaussian. The green channel has been reported to show the highest contrast between the blood vessels and background [22]. A steerable first order derivative of Gaussian filter is used to implement the kernel [23]. The steerable filter has high responses to the gradient along different angles at different locations. The separable first order derivative of Gaussian along the x-axis and along the y-axis are given by 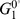 and 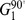. The first order derivative of Gaussian along any angle 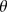 is defined in Equation 5.
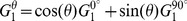
(5)The original image is first convolved with 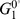 and 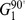 to get the first order derivative image along the x-axis and y-axis. Then within each normal profile, the weights of the profile nodes are calculated according to equation 5. In our implementation, angle 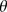 is the graph column direction, i.e., the direction of the electric lines of force at each graph node location.

### Graph Search and Boundary Determination

For each branch point, the three boundaries are constructed as three independent single slice graphs. Intra-column smoothness constraints are set to maintain the smoothness within the slice. After the graphs are built, the optimal segmentation is found and the vessel boundary is determined as described by Li *et al.* [24].

Once the three boundaries are determined, the vessel width is measured as the Euclidean distance between the graph nodes on the segmentation from the same centerline pixel, as illustrated in Figure 3 (c).

### Experimental Methods

**Figure 4:**
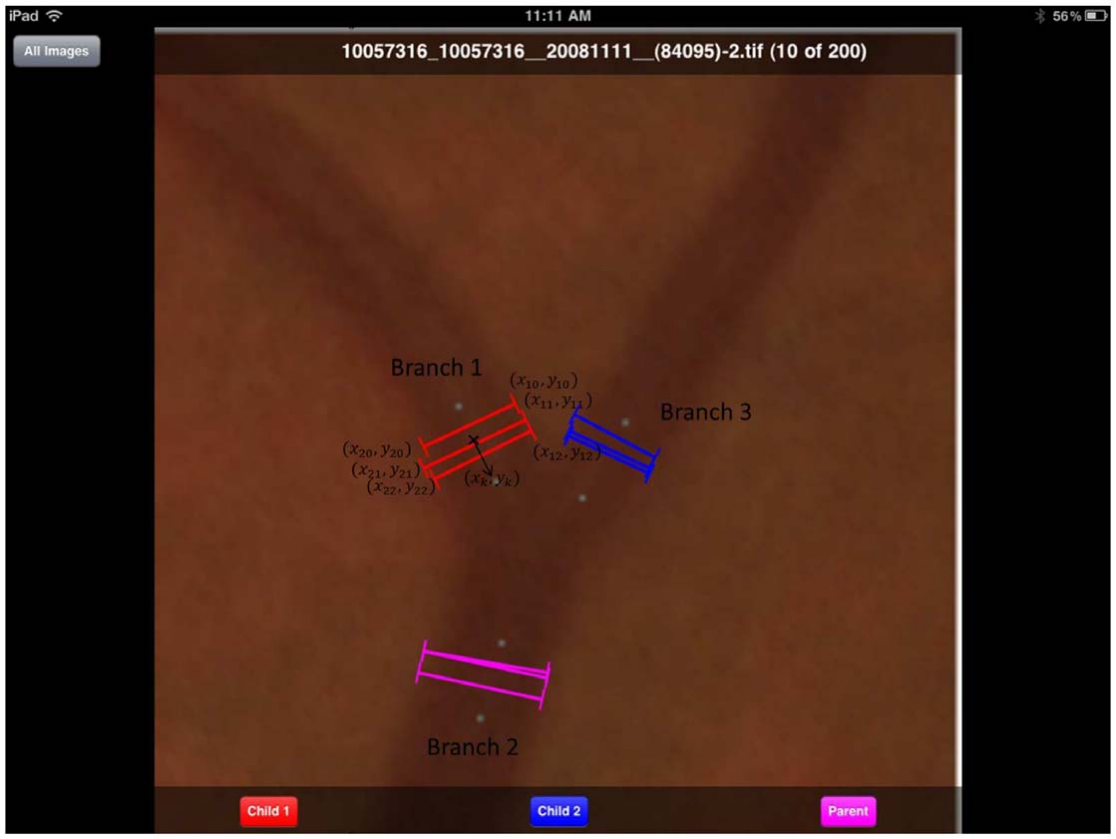
An illustration of the human expert annotation. Blue dots denote the region where the measurement should be given. Black text were superimposed by the author for the sake of illustration. Vessel width for branch 1 is calculated as the average of the three width profiles. The branch center for branch 1 is calculated as the average of the three width profile center.

A set of 100 artery branchings and 100 vein branchings were selected from 50 fundus images from 50 normal subjects and used to assess vessel width measurement performance (available on INSPIRE website: http://webeye.ophth.uiowa.edu/component/k2/item/270). These 50 normal subjects were selected randomly from the Tromsø study cohort. The Tromsø study was initiated in 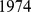 in an attempt to help combat the high mortality of cardiovascular diseases in Norway. The Tromsø study consists of six surveys that have been conducted in the municipality of Tromsø from 1974 to 2008. This population study includes 40051 subjects in total who have attended at least on of the six surveys. There are currently some 100 different ongoing research projects based on the data from the consecutive six surveys. A good overview of the Tromsø study is in Jacobsen et al [25]. All subjects provided written informed consent for participation in this study, and all images were de-identified before sharing with the University of Iowa. The research team at the University of Iowa did not have access to any patient identifiable information, and the study was therefore declared exempt by the institution review board of the University of Iowa. All research was in accordance with the tenets of the Declaration of Helsinki. The image resolution is 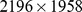 pixels, and images were stored in DICOM format. A pixel is approximately 3.7 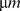 on each side. For each fundus image, two artery branches and two vein branches were initially selected (based on their contrast) to evaluate the algorithm. In order to make sure the measurements given by experts and algorithm are comparable, i.e., at approximately the same location, for each branch, a start point and an end point were given to indicate where the measurements should be performed. For each branch, the start point was at approximately one vessel diameter (15 pixels) away from the branch center. The end point was at approximately two vessel diameters away from the branch center. The three start points and three end points are shown in Figure 4 as blue dots on the branch.

The two experts are denoted as 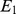 and 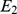 respectively. The manual measurements were performed on a tablet-based color fundus image evaluation system ([26]) and the interface is given in Figure 4. The blue dots denote the region within which the measurement should be given. The black texts were superimposed by the author for the sake of illustration. Each expert was told to annotate at least three measurement profiles to each of the three branches for each selected branch point. Each measurement profile contains seven outputs: image name, image index, branch index, and profile start and end locations 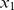, 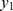, 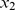, and 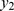. The branch center is defined as 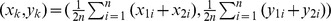 and the width of the branch is defined as 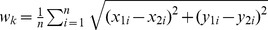, where 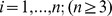 is the number of measurement at the given branch and 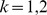 denotes the two experts.

## Results

**Figure 5:**
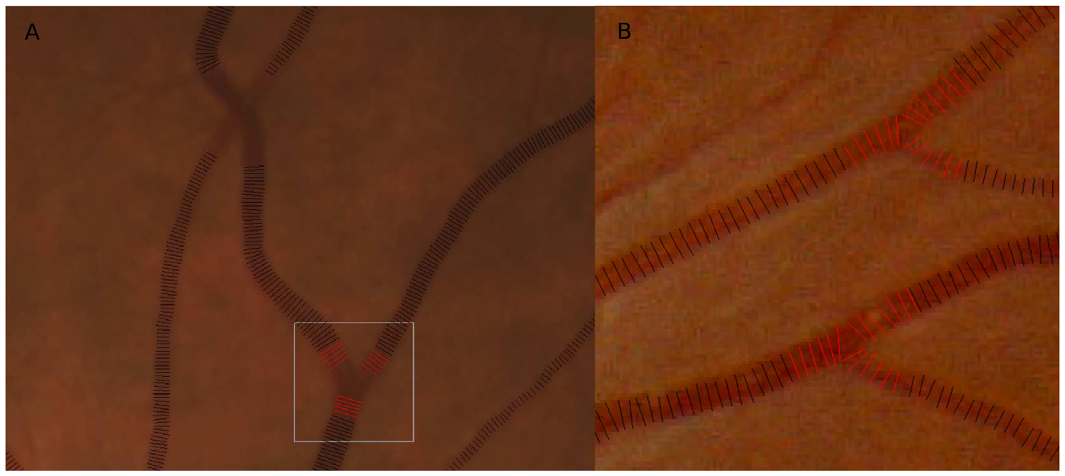
Typical vessel width measurement images, with red lines denoting the vessel width measurement for branch points and black lines denoting the vessel width measurement for straight vessels. (a) shows the vessel width measurement of Figure 1.

Typical vessel width measurements are shown in Figure 5. Red lines denote the vessel width measurement for branch points. Black lines denote the vessel width measurement for straight vessels.

We compared the performance of our algorithm with the performance of the two human experts. As mentioned, the branch center is defined as 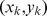, where 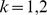 are the two experts. Similarly, we determine the branch center calculated in our algorithm as 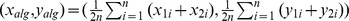, where 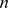 is the number of measurement profiles given by the algorithm. When the performance is compared, 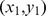, 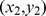, 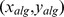 are matched. A match is considered successful if the Euclidean distance between the two centers is less than 15 pixels (approximately one vessel diameter). The match could fail when 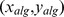 is too far from either 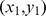 or 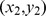, or 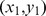 and 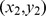 are too far from each other. The performance for a branching is considered as valid if the matches of all three branches are successful. Branchings were detected automatically. Among the 200 branchings, 3 out of 200 expert selected branchings were not detected by our automatic approach and were not included in further analysis. Hence, 197 out of 200 (98.5%) of the branches were used for further vessel width comparison. The vessel width is compared for valid branchings. The average of 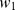 and 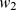 is considered as the ground truth for comparison and is denoted as AVE in Table 1, which shows the signed errors and unsigned errors. The result of the comparison is given in Table 2. 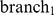, 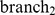 and 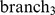 are the three branches.

**Table 1:** Comparison of the performance between human experts and presented method (signed error and unsigned error in pixels).

		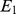 vs. 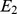			Alg vs. Ave		
		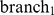	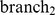	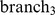	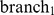	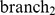	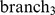
Signed Error	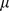	−1.160	−1.440	−1.026	−0.408	−0.532	−0.102
	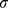	1.557	1.882	−1.932	−1.670	−1.895	−2.090
Unsigned Error	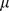	1.544	1.884	1.695	1.253	1.480	1.534
	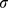	1.176	1.435	1.378	1.174	1.294	1.419

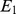
 and 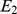 denote the two human experts. 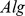 denotes the presented method. 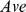 denotes the average measurements of the two human experts. 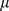 and 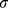 are the mean and standard deviation of errors.

**Table 2:** Comparison of the performances between human experts and presented method (vessel width in pixels).

	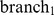		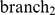		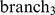	
	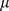	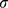	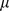	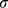	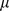	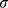
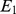	18.24	4.82	14.57	4.53	13.37	3.66
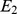	19.40	4.94	16.01	4.34	14.39	3.52
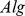	18.41	4.81	14.75	4.20	13.78	3.69

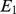
 and 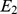 denote the two human experts. 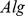 denotes the presented method. 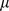 and 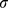 are the mean and standard deviation of vessel width measurements.

**Figure 6:**
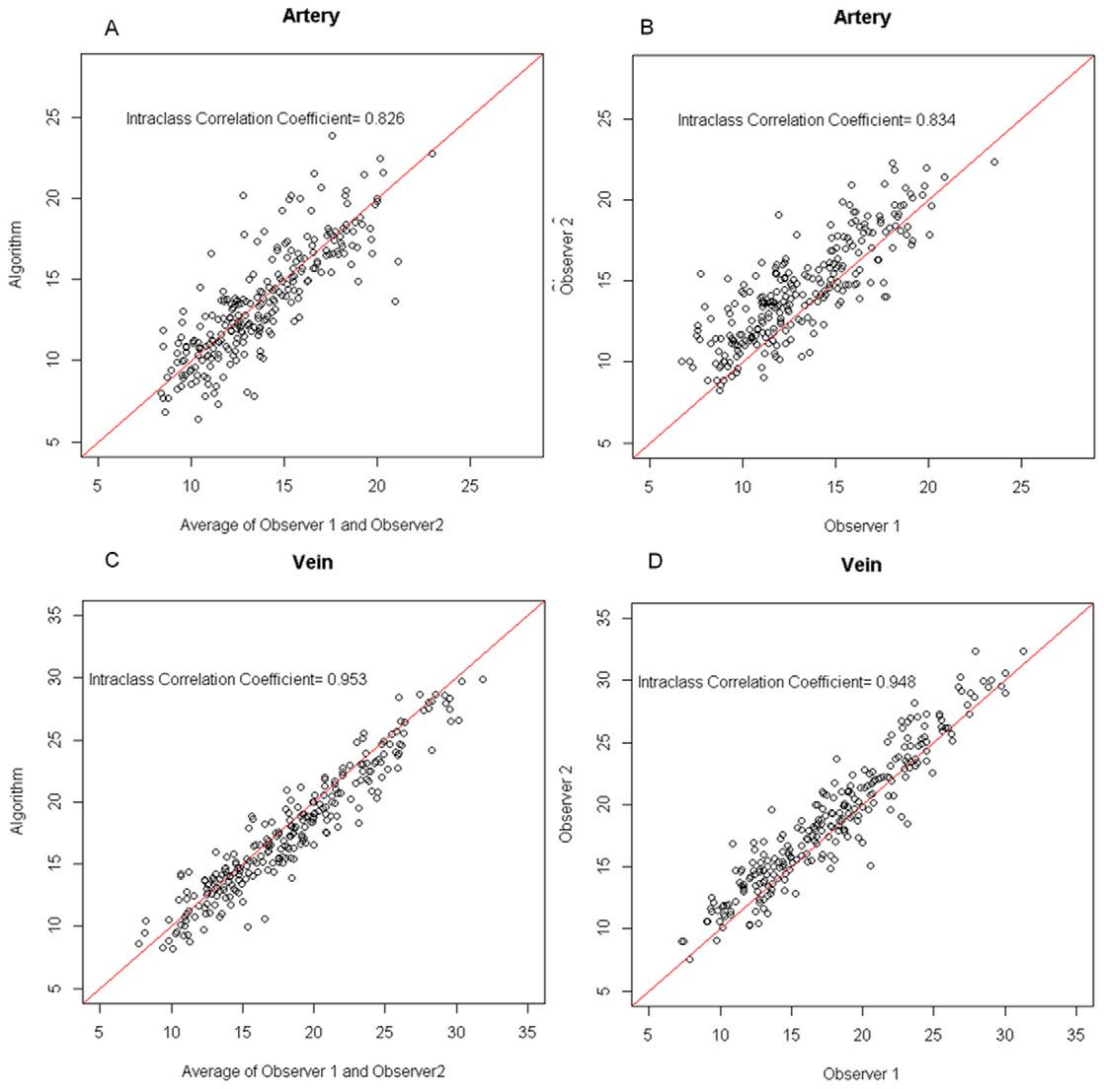
Vessel width measurement scatter plots in pixel. (a) The scatter plot of vessel width measured by automatic algorithm and the average of vessel width given by expert 1 and expert 2 for artery branchings. (b) The scatter plot of vessel width measurement performed by expert 1 and expert 2 for artery branchings. (c) The scatter plot of vessel width measured by automatic algorithm and the average of vessel width given by expert 1 and expert 2 for vein branchings. (d) The scatter plot of vessel width measurement performed by expert 1 and expert 2 for vein branchings.

The scatter plots are given in Figure 6. Arteries and veins were plotted separately. The Intraclass Correlation Coefficient is given to quantify how consistent it is between different measurements. The measurements for venous branches showed a better consistency both between experts, and between experts and the automatic algorithm.

**Figure 7:**
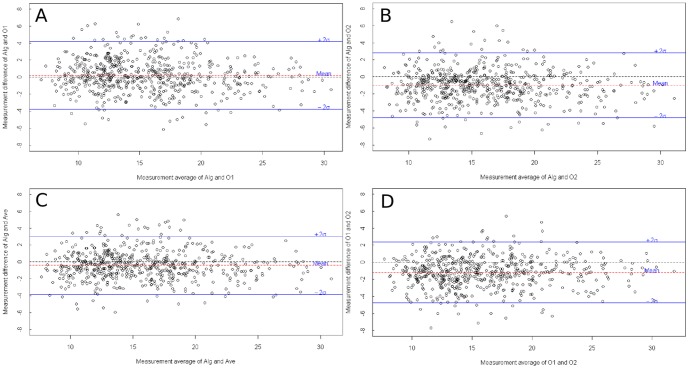
Bland-Altman plots, in pixel. (a) Bland-Altman plot of proposed method and expert 1. (b) Bland-Altman plot of proposed method and expert 2. (c) Bland-Altman plot of proposed method and the average of expert 1 and expert 2. (d) Bland-Altman plot of expert 1 and expert 2.

The Bland-Altman plots are given in Figure 7.

## Discussion

**Figure 8:**
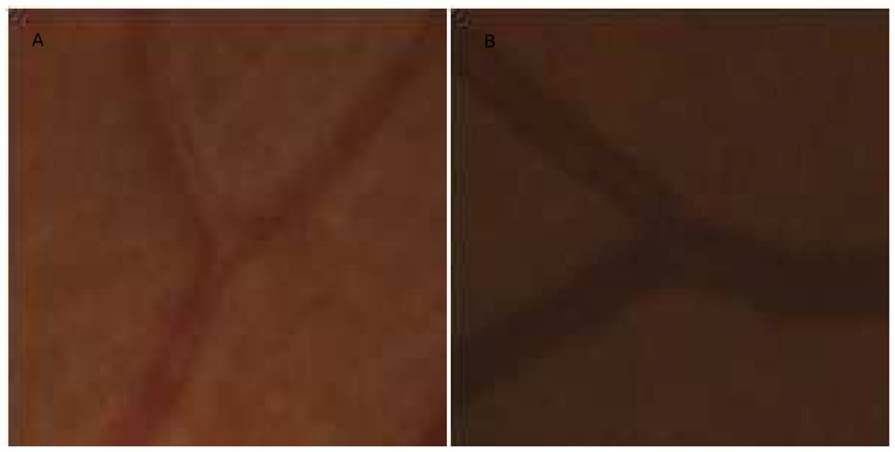
Examples of typical arterial branch point and venous branch point. Arteries usually have a lower contrast to the background comparing with veins. (a) A typical arterial branch point. (b) A typical venous branching.

We developed and validated a fully automatic vessel width measurement method for branch points on retinal images. Performance of the method was comparable to the performance of human experts. Both the human experts and the automatic method showed a lower performance for artery compared to vein branch points. We observed that the veins usually have a more clearly defined vessel boundary than arteries, because of the increased absorption of light by de-oxygenated (venous) hemoglobin versus oxygenated (arterial) hemoglobin. For instance, Figure 8 shows a typical artery branch and a typical vein branch. This might have resulted in the ambiguities in arterial vessel width measurement for both human experts and the proposed automatic method.

The proposed method is computationally efficient. For a retinal image of 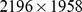 pixels in size, the vessel segmentation map generation and image centerline generation takes less than 10 seconds. The average number of straight vessel segments on the image is 90 and the number of branching is about 30. It takes around 17 seconds to solve the graph for straight vessels and about 9 seconds to solve the graph for branch points.

The method suffers from the limitation that it relies largely on the quality of the vessel segmentation and centerline image. If the vessel centerline image exhibits a false positive branch point, the algorithm will still try to delineate the vessel boundaries, resulting in invalid measurements. However, our algorithm is not dependent on any specific vessel segmentation approach, and therefore, as improved vessel segmentations become available, our algorithm can be expected to improve as well.

An important application of the vessel width relationship at branch points is in artery-to-vein ratio (AVR). In the area of retinal image analysis, AVR is an important parameter, associated with stroke and other cardiovascular events in adults, and associated with an increased risk of retinopathy of prematurity in premature infants [7]. AVR is defined as the ratio between *Central Retinal Artery Equivalent* (CRAE) and *Central Retinal Vein Equivalent* (CRVE) [15], [16]. The vessel width relationship at arterial branch points and venous branch points is the most important information in AVR calculation. However, until now, only manual or semi-automatic methods are available to measure this relationship [1]–[4],[11],[16]. Due to the laboriousness of manual and semi-automatic work, only a limited number of measurements have been available to estimate the relationship, which reduced the reliability of the measurement. Future work of this study includes the application of the proposed method in AVR calculation.

## Conclusions

We proposed a retinal vessel width measurement method at branch points based on an improved electric field theory motivated graph approach. This first fully automatic retinal branching vessel width measurement method has a performance comparable to human experts.

## References

[1] Doubal FN, de Haan R, MacGillivray TJ, Cohn-Hokke PE, Dhillon B, et al (2010) Retinal arteriolar geometry is associated with cerebral white matter hyperintensities on magnetic resonance imaging. International Journal of Stroke 5: 434–439. 21050397 10.1111/j.1747-4949.2010.00483.x PMC3489044

[2] Parr JC, Spears GFS (1974) Mathematic Relationships Between the Width of A Retinal Artery and The Width of Its Branches. American Journal of Ophthalmology 77: 478–483. 4819452 10.1016/0002-9394(74)90458-9

[3] Chapman N, Dell'omo G, Sartini MS, Witt N, Hughes A, et al (2002) Peripheral vascular disease is associated with abnormal arteriolar diameter relationships at bifurcations in the human retina. Clinical Science 103: 111–116. 12149100 10.1042/cs1030111

[4] Stanton AV, Wasan B, Cerutti A, Ford S, Marsh R, et al (1995) Vascular Network Changes in the Retina width Age and Hypertension. Journal of Hypertension 13: 1724–1728. 8903640

[5] Abràmoff MD, Garvin MK, Sonka M (2010) Retinal Imaging and Image Analysis. IEEE Reviews in Biomedical Engineering 3: 169–208. 22275207 10.1109/RBME.2010.2084567 PMC3131209

[6] Patton N, Aslam TM, Macgillivray T, Pattie A, Deary IJ, et al (2005) Retinal vascular analysis as a potential screening tool for cerebrovascular disease: a retionale based on homology between cerebral and retinal microvasculatures. Journal of Anatomy 206: 319–348. 15817102 10.1111/j.1469-7580.2005.00395.x PMC1571489

[7] Sun C, Wang JJ, Mackey Da, Wong TY (2003) Retinal Vascular Caliber: Systemic, Environmental, and Genetic Associations. Survey Ophthalmol 48: 245–255. 10.1016/j.survophthal.2008.10.003 19171211

[8] Tsai CL, Stewart CV, Tanenbaum HL, Roysam B, Steward CV, et al (2004) Model-based method for improving the accuracy and repeatability of estimating vascular bifurcations and crossovers from retinal fundus images. IEEE Transactions on Information Technology in BioMedicine 8: 122–130. 15217257 10.1109/titb.2004.826733

[9] Bhuiyan A, Nath B, Chua J, Ramamohanarao K (2008) Automatic detection of vascular bifurcations and crossovers from color retinal fundus images. In: International IEEE Conference on Signal-Image Technologies and Internet-Based System. IEEE, pp. 711–718.

[10] Shen H, Roysam B, Steward CV, Turner JN, Tanenbaum HL, et al (2001) Optimal scheduling of tracing computations for real-time vascular landmark extraction from retinal fundus images. IEEE Transactions on Information Technology in BioMedicine 5: 77–91. 11300219 10.1109/4233.908405

[11] Patton N, Aslam T, MacGillivray T, Dhillon B, Constable I (2006) Asymmetry of Retinal Arteriolar Branch Widths at Junctions Ability of Formulae to Predict Trunk Arteriolar Widths. Investigative Ophthalmology & Visual Science 47: 1329–1333. 16565364 10.1167/iovs.05-1248

[12] Quelhas P, Boyce J (2003) Vessel Segmentation and Branching Detection using an Adaptive Profile Kalman Filter in Retinal Blood Vessel Structure Analysis. In: Conference on Pattern Recognition and Image Analysis, ibPRIA. Springer-Verlag LNCS, pp. 802–809.

[13] Calvo D, Ortega M, Penedo MG, Rouco J (2011) Automatic detection and characterisation of retinal vessel tree bifurcations and crossovers in eye fundus images. Computer methods and programs in biomedicine 103: 28–38. 20643492 10.1016/j.cmpb.2010.06.002

[14] Lee S, Reinhardt JM, Cattin PC, Abràmoff MD (2010) Objective and Expert-independent validation of retinal image registration algorithms by a projective imaging distortion model. Medical Image Analysis 14: 539–549. 20493760 10.1016/j.media.2010.04.001

[15] Hubbard LD, Brothers RJ, King WN, Clegg LX, Klein R, et al (1999) Methods for evaluation of retinal Microvascular abnormalities associated with hypertension/sclerosis in the atherosclerosis risk in communities study. Ophthalmology 106: 2269–2280. 10599656 10.1016/s0161-6420(99)90525-0

[16] Knudtson MD, Lee KE, Hubbard LD, Wong TY, Klein R, et al (2003) Revised formulas for sum-marizing retinal vessel diameters. Current Eye Research 27: 143–149. 14562179 10.1076/ceyr.27.3.143.16049

[17] Chapman N, Witt N, Gao X, Bharath AA, Stanton AV, et al (2001) Computer algorithms for the automated measurement of retinal arteriolar diameters. British Journal of Ophthalmology 85: 74–79. 11133716 10.1136/bjo.85.1.74 PMC1723694

[18] Xu X, Niemeijer M, Song Q, Sonka M, Garvin MK, et al (2011) Vessel boundary delineation on fundus images using graph-based approach. IEEE Transactions on Medical Imaging 30: 1184–1191. 21216707 10.1109/TMI.2010.2103566 PMC3137950

[19] Niemeijer M, Staal J, van Ginneken B, Loog M, Abramoff MD (2004) Comparative study of Retinal Vessel Segmentation Methods on a New Publicly Available Database. In: SPIE Medical Imaging. Spie, volume 5370, pp. 648–656.

[20] Sonka M, Hlavac V, Boyle R (1998) Image Processing, Analysis, and Machine Vision. New York: Thomson Learning, 3rd edition.

[21] Yin Y, Song Q, Sonka M (2009) Electric Field theory motivated graph construction for optimal medical image segmentation. In: Graph Based Representations in Pattern Recognition. Springer, pp. 334–342.

[22] Lee S, Abràmoff MD, Reinhardt JM (2010) Retinal atlas statistics from color fundus images. In: SPIE Medical Imaging. volume 7623.

[23] Freeman WT, Adelson EH (1991) The Design and Use of Steerable Filters. IEEE Transactions on Pattern Analysis and Machine Intelligence 13: 891–906.

[24] Li K, Wu X, Chen DZ, Sonka M (2006) Optimal Surface Segmentation in Volumetric Images-A Graph Theoretic Approach. IEEE Transactions on Pattern Analysis and Machine Intelligence 28: 119–134. 16402624 10.1109/TPAMI.2006.19 PMC2646122

[25] Jacobsen BK, Eggen AE, Mathiesen EB, Wilsgaard T, Njø lstad I (2011) Cohort profile: The Tromso Study. International Journal of Epidemiology : 1–7. 10.1093/ije/dyr049 PMC3429870 21422063

[26] Christopher M, Moga DC, Russell SR, Folk JC, Scheetz T, et al (2012) Validation of Tablet-Based Evaluation of Color Fundus Images. Retina 0: 1–7. 10.1097/IAE.0b013e3182483361 PMC3396716 22495326

